# The Association and Pathogenesis of SERPINA3 in Coronary Artery Disease

**DOI:** 10.3389/fcvm.2021.756889

**Published:** 2021-12-08

**Authors:** Bo Li, Zhijun Lei, You Wu, Bingyu Li, Ming Zhai, Yuan Zhong, Peinan Ju, Wenxin Kou, Yefei Shi, Xianling Zhang, Wenhui Peng

**Affiliations:** Department of Cardiology, Shanghai Tenth People's Hospital, School of Medicine, Tongji University, Shanghai, China

**Keywords:** SERPINA3, CAD, atherosclerosis, RASMCs, HUVECs

## Abstract

**Background:** Serine proteinase inhibitor A3 (SERPINA3) has been discovered in the pathogenesis of many human diseases, but little is known about the role of SERPINA3 in coronary artery disease (CAD). Therefore, we aim to determine its relationship with CAD and its function in the pathogenesis of atherosclerosis.

**Methods:** In total 86 patients with CAD and 64 patients with non-CAD were compared. The plasma SERPINA3 levels were measured using ELISA. Logistic regression analysis and receiver-operating characteristic (ROC) analysis were performed to illustrate the association between plasma SERPINA3 levels and CAD. In vitro, real-time PCR (RT-PCR) and immunofluorescence staining were used to determine the expression of SERPINA3 in atherosclerotic plaques and their component cells. Then rat aortic smooth muscle cells (RASMCs) were transfected with siRNA to knock down the expression of SERPINA3 and human umbilical vein endothelial cells (HUVECs) were stimulated by SERPINA3 protein. EdU assay and scratch assay were used for assessing the capability of proliferation and migration. The cell signaling pathway was evaluated by western blot and RT-PCR.

**Results:** Patients with CAD [104.4(54.5–259.2) μg/mL] had higher levels of plasma SERPINA3 than non-CAD [65.3(47.5–137.3) μg/mL] (*P* = 0.004). After being fully adjusted, both log-transformed and tertiles of plasma SERPINA3 levels were significantly associated with CAD. While its diagnostic value was relatively low since the area under the ROC curve was 0.64 (95% CI: 0.55–0.73). Secreted SERPINA3 might increase the expression of inflammatory factors in HUVECs. Vascular smooth muscle cells had the highest SERPINA3 expression among the aorta compared to endothelial cells and inflammatory cells. The knockdown of SERPINA3 in RASMCs attenuated its proliferation and migration. The phosphorylated IκBα and its downstream pathway were inhibited when SERPINA3 was knocked down.

**Conclusions:** Elevated plasma SERPINA3 levels were associated with CAD. SERPINA3 can increase inflammatory factors expression in HUVECs. It can regulate VSMCs proliferation, migration, and releasing of inflammatory factors through the NF-κB signaling pathway. Thus, SERPINA3 played a significant role in the pathogenesis of atherosclerosis.

## Introduction

Coronary artery disease (CAD) remains the leading cause of mortality and morbidity worldwide, despite rapid advances in pharmacological and interventional treatment strategies (1). It is well-accepted that atherosclerosis is the most common cause of CAD. Traditional cardiovascular risk factors including diabetes mellitus (DM), hypertension, dyslipidemia, smoking, and obesity can lead to inflammation and initiation of atherosclerosis. Besides, growing evidence shows that atherosclerosis is an inflammatory disease and inflammation plays a vital role in the atherosclerotic process (2). Pathologically, caused by the entry and retention of lipid in the subendothelial space, complex inflammatory responses orchestrate the progression and outcome of the disease. Different cells, including endothelial cells, smooth muscle cells, macrophages and granulocytes, and inflammatory cytokines form an inflammatory microenvironment, play a vital role in the initiation and progression of plaque (3, 4). In addition, the accumulation of abundant inflammatory cells promotes plaque rupture and affects plaque stability (5). Hence, inflammation plays a pivotal part in all stages of atherosclerosis, from plaque formation to eventual plaque rupture (6).

Serpins are a superfamily of proteins that act as serine protease inhibitors. Serpins engage in many essential processes such as oxidative stress and the inflammatory response. *In vivo* and *in vitro* studies have demonstrated that some serpins are associated with cardiovascular disease (7–9). Our previous work revealed that SERPINA12, also known as Vaspin (visceral adipose tissue-derived serpin), is decreased in patients with CAD and correlated to the severity of CAD (10). We also found that SERPINA12 may be a valuable predictor for major adverse cardiovascular events (MACE) in patients with acute myocardial infarction (AMI) and chest pain (11, 12).

Serine proteinase inhibitor A3 (SERPINA3), also named antichymotrypsin (ACT), is another member of the Serpin superfamily. Several serine proteases, such as leukocyte cathepsin G, mast cell chymases, pancreatic chymotrypsin, and lung serum protease, are inhibited by SERPINA3 (13). Moreover, cathepsin G expressed in neutrophil granules and secreted by inflammation is the primary target for SERPINA3 (13). SERPINA3 is a typical acute-phase protein, and the plasma SERPINA3 level increases dramatically during inflammation (14, 15). Existing data show that SERPINA3 is involved in the pathogenesis of chronic obstructive pulmonary disease, Parkinson's disease, Alzheimer's disease, and cancer (13). An increased local expression of SERPINA3 was found in atherosclerotic lesions (16). Zhao et al. reported that circulating SERPINA3 levels may be a potential predictor of MACE in AMI. However, the association between plasma SERPINA3 levels and CAD and the mechanism of SERPINA3 in the progression of atherosclerosis are still unknown. Hence, we conducted a clinical study to explore the relationship between plasma SERPINA3 levels and CAD and experimental research to reveal the role of SERPINA3 in the pathogenesis of atherosclerosis.

## Materials and Methods

### Patient Study

This study enrolled 86 patients with CAD diagnosed by coronary angiography (CAG), of whom 74 (86.0%) had stable CAD and 12 (14.0%) had acute coronary syndrome (ACS). These patients were compared with 64 non-CAD patients confirmed by CAG. The exclusion criteria are patients with any previous ischemic stroke or CAD, acute pulmonary embolism, acute or chronic heart failure, valvular heart disease, severe hepatic disease or renal disease, acute infection, and cancer. CAD was defined as ≥50% luminal diameter stenosis of at least one major epicardial coronary artery. The severity of CAD was determined by the number of vessels with significant lesions and SYNTAX score II (http://www.syntaxscore.com/calculator/start.htm). The stable CAD and ACS were diagnosed according to the ESC guidelines (2020) (17). Demographic characteristics, comorbidities, cardiovascular risk factors and laboratory tests data were collected and checked by two doctors. All participants provided written informed consent and the study was approved by the Shanghai Tenth People's Hospital's Ethics Committee.

### ELISA Assays and Biochemical Investigations

Blood samples were collected in EDTA-containing tubes after a 10-h overnight fast and centrifuged at 4°C, 3,000 g for 10 min, then serum specimens were stored at −20°C for analysis. Plasma SERPINA3 levels were measured by ELISA kit (AACT Human ELISA Kit, Cat#ab157706, Abcam) according to the manufacturer's instructions.

### Animal Study

All animal studies were conducted according to the Guide for the Care and Use of Laboratory Animals (U.S. National Institutes of Health (NIH) Bethesda, MD, USA), and were handled humanely under animal experiment protocols approved by the Animal Care and Use Committee of Shanghai Tenth People's Hospital. Six-week-old male wild-type mice and *Ldlr*^−/−^ male mice were generated by GemPharmatech and fed with a western diet (Cat# XT108C, Jiangsu Xietong) for 20 weeks. Each group contained five mice. All mice were bred with a C57BL/6 background and housed in the SPF laboratory animal room with daylight from 7 a.m. to 7 p.m.

A male Sprague–Dawley rat weighing nearly 220 g was generated by Shanghai Sippe-Bk Lab Animal Co., Ltd. and used to extract rat aortic smooth muscle cells (RASMCs).

### Cell Culture

Human umbilical vein endothelial cells (HUVECs) and human aortic smooth muscle cells (HASMCs) were purchased from ScienCell Research Laboratories (Cat#8000, #6110, ScienCell), HUVECs were cultured in endothelial cells medium (Cat#1001, ScienCell) with 5% fetal bovine serum (FBS), 1% endothelial cells growth supplement (ECGS), and 1% penicillin/streptomycin; HASMCs were cultured in smooth muscle cell medium (SMCM, Cat. #1101, ScienCell, USA) with 2% FBS (Cat#1600-0044, Gibco), 1% SMCGS, and 1% penicillin and streptomycin. Experiments were performed using cells from passages 2–8. Human myeloid leukemia mononuclear cells (THP-1) obtained from Fudan University Institutes of Biomedical Sciences Cell Center (Shanghai, China) were cultured in Dulbecco's Modified Eagle Medium (DMEM, Cat#ZQ-100, Shanghai Zhong Qiao Xin Zhou Biotechnology) with 10% FBS and 1% penicillin/streptomycin. Isolation of RASMCs from the thoracic aortas of male Sprague–Dawley rats were performed as previously described (18). Cells were cultured in DMEM with 20% FBS and 1% penicillin/streptomycin. All types of cells were incubated in 5% CO_2_ at 37°C.

### SiRNA Transfection and Cell Stimulation

RASMCs seeded to 6 or 12-well dishes were transfected by siRNA targeting SERPINA3 and scramble siRNA (siCtr) (Genepharma) at about 70–80% confluency, by using Lipofectamine® RNAiMAX reagent (Invitrogen) according to the user manual. After 1 day, transfected cells were stimulated by ox-LDL (50 μg/mL, Cat#sc-7950002, Santa Cruz) for 12 h. HUVECs were stimulated with ox-LDL (100 μg/mL) and human recombinant SERPINA3 protein (the following text is written as SERPINA3, 100 ng/mL, Cat#ag2830, Proteintech) for 18 h. All cells were then used for the following experiments.

### Cell Proliferation Analysis

Cell proliferation was assessed by 5-ethynyl-2′-deoxyuridine (EdU) incorporation assay (Cat#40276ES60, Yeasen, Shanghai). RASMCs and HUVECs in 12-well plates were washed with PBS and incubated with an EdU-labeling mixture (10 mM) for 12 h. Cells then were fixed, permeabilized, and EdU incorporation was detected according to the manufacturer's instructions. Images were captured by fluorescence microscope (Olympus, Japan). Data were presented as a ratio of EdU-positive cells to total cells.

### Scratch Assay

Scratch assay was used to detect cells' migratory activity. After transfection and stimulation, RASMCs and HUVECs in 12-well plates went through a 12 h starvation and then were scraped by a 200 μl pipette tip across the center of the well. The scratch assays were monitored at 0, 12, 24, and 36 h after wounding.

### RNA Isolation and Real-Time Quantitative RT-PCR

RNA was isolated by RNA Purification Kit (Cat#RN001, EZBioscience) and reverse transcribed by HiScript III RT SuperMix reverse-transcription reagent kit (Cat#711-02/03, Vazyme Biotech Co.). qPCR was performed on a LightCycler 96 Real-Time PCR System (F. Hoffmann-La Roche) using FastStart Essential DNAGreen Master assay (F. Hoffmann-LaRoche). The primers used are listed in Supplementary Table 1. All of the primers were synthesized by Sangon, Shanghai.

### Western Blot Analysis

RASMCs after treatment were lysed by 1 × cell lysis (Cat#9803, Cell Signaling Technologies) with protease inhibitors (Cat#04693159001, Roche Molecular Biochemicals, USA) and used for western blot. Lysates were centrifuged at the speed of 12,000 g at 4°C for 10 min. The supernatants were quantified using bicinchoninic acid kit (Cat#20201ES76, Yeasen) and subjected to SDS-polyacrylamide gel electrophoresis and electro-transferred onto PVDF membranes. Membranes were blocked with 5% BSA in PBST for 1 h prior to overnight incubation with the indicated primary antibodies, followed by incubation with fluorescent secondary antibodies diluted in PBST for 1 h at room temperature in the dark. HRP was detected using the Super Signal chemiluminescence reagent substrate (Cat#32134, Thermo Fish Scientific). Western blot was imaged with the Biorad Chemdoc system according to protocol instructions. Primary antibodies for SERPINA3 (Cat#A1021, Abcolonal), total and phosphorylated IκBα (Cat#92425S, 2859S, Cell Signaling Technology), PCNA (Cat#4502103, sigma), Cyclin D1 (Cat#2922S, Cell Signaling Technology) and vinculin (Cat#sc-73614, Santa Cruz) were all at a concentrate of 1:1000.

### Immunofluorescence Staining

Mice aortic tissues were harvested and processed in optimal cutting temperature compound and sliced into 5 μm-thick sections. Cryosections were incubated with anti-SERPINA3 (1:200), anti-α-smooth muscle actin (anti-α-SMA, Cat#ab7817, Abcam, 1:200) overnight at 4–°C. Normal isotype IgG (Cat#sc-2025, Santa Cruz) was used as a negative control. After washing with phosphate-buffered saline (PBS), secondary antibodies (Alexa Fluor 594-conjugated goat anti-mouse and Alexa Fluor 488-conjugated goat anti-rabbit, Thermo Fisher Scientific, 1:200) were incubated for 1 h at 37°C in the dark. Nuclei were labeled with DAPI (Vector Laboratories), and the images of cryosections were taken by Leica DMI6000 microscopy.

### Statistical Analysis

In the clinical study, normal distribution data were compared by Student's *t*-test and displayed as mean ± standard deviation (SD). Skewed data were compared by the Mann-Whitney *U*-test and expressed as median (25th−75th). The categorical variables were compared by the chi-square or Fisher exact tests and presented as frequencies and percentages. Plasma SERPINA3 levels were log-transformed due to skewed distribution. Correlation analysis was performed using Spearman's correlation. Univariate and multivariate logistic regression analysis was conducted to determine the association between plasma SERPINA3 levels and CAD. The receiver-operating characteristic (ROC) curves were performed to clarify the diagnostic value of SERPINA3. All experiment data were presented as mean ± SD. Student-s *t*-test, two-way ANOVA followed by Bonferroni's *post-hoc* test were performed for statistical analysis. All graphs were generated from a minimum of three experiments.

All tests were two-sided and *P* < 0.05 was considered significant. All data analyses were completed by SPSS22.0 (SPSS Inc., Chicago, IL, USA) and GraphPad Prism 9.0.

## Results

### Baseline Characteristics of Patient Study

Supplementary Table 2 showed the demographic and laboratory characteristics of patients in CAD and non-CAD groups. Patients with CAD had a male predominance over patients without CAD (67.4 vs. 35.9%, *P* < 0.001). Past medical histories, including DM (24.4 vs. 9.4%, *P* = 0.018) and smoking (32.6 vs. 14.1%, *P* = 0.009) were more frequent in the CAD group than in the non-CAD group. Besides, patients from CAD group had lower levels of high-density lipoprotein cholesterol (HDL-C) [(1.03(0.86–1.27) mmol/L vs. 1.13(0.97–1.39) mmol/L, *P* = 0.011], higher levels of fasting blood glucose [5.3(4.7–6.5) mmol/L vs. 4.9(4.6–5.4) mmol/L, *P* = 0.012] and glycated hemoglobin A1c (HbA1c) [6.3% (5.8–7.0%) vs. 6.1% (5.8–6.4%), *P* = 0.042].

### Association Between Plasma SERPINA3 Levels and Inflammation and Metabolism-Related Parameters

**Figure 1:**
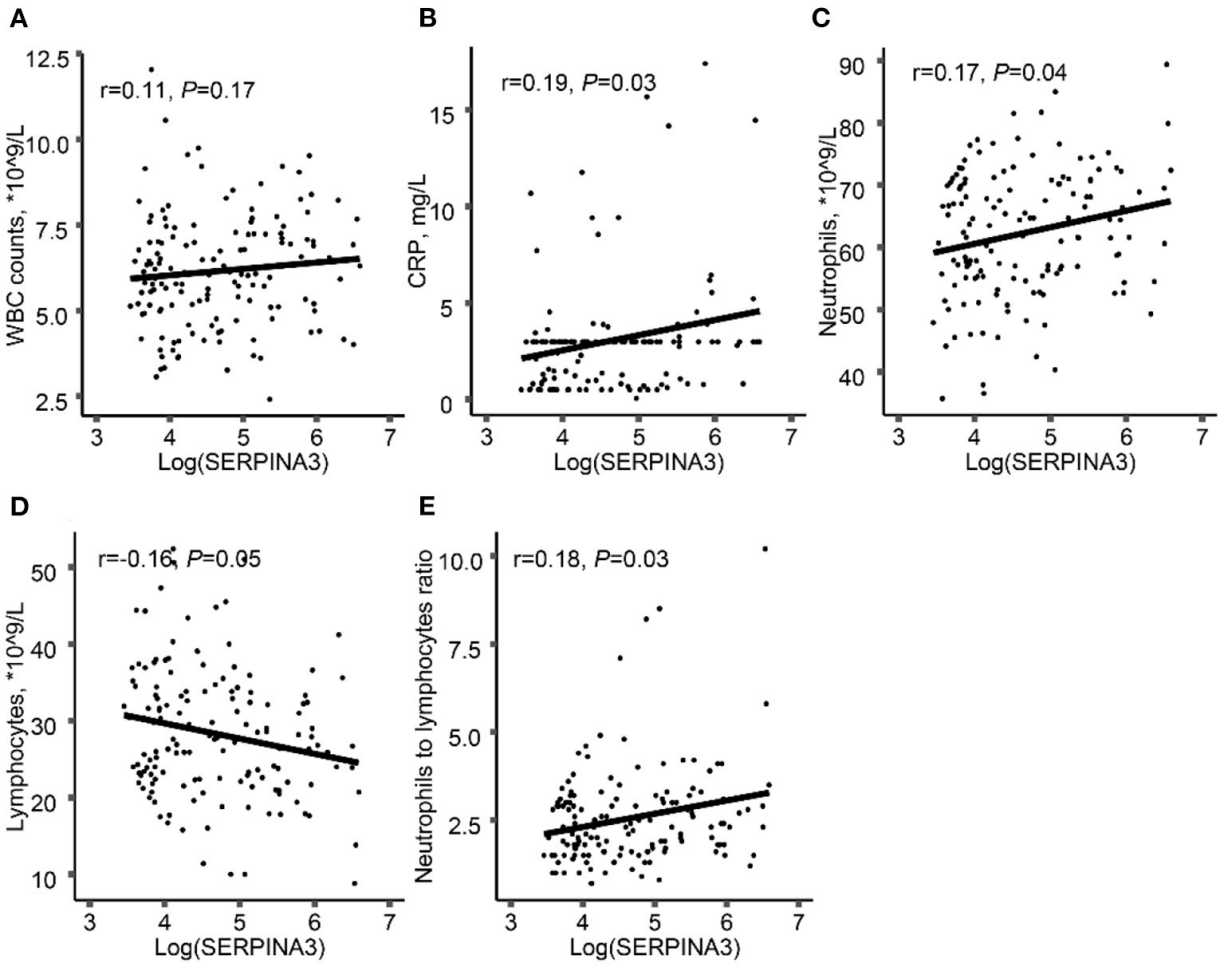
Associations between log-transformed plasma SERPINA3 levels and inflammation-related parameters. Associations between log-transformed plasma SERPINA3 levels and WBC counts **(A)**, CRP **(B)**, neutrophils **(C)**, lymphocytes **(D)**, and NLR **(E)**. WBC, white blood cell; CRP, C-reactive protein; NLR, neutrophils to lymphocytes ratio.

Considering SERPINA3 is an acute-phase protein during inflammation, we found that there was a positive correlation between log-transformed plasma SERPINA3 levels and CRP (Figure 1B), neutrophils count (Figure 1C), and neutrophil-to-lymphocyte ratio (NLR) (Figure 1E). However, no relationships between log-transformed plasma SERPINA3 levels and white blood cells (Figure 1A) and lymphocytes counts (Figure 1D) were observed. We also found that log-transformed plasma SERPINA3 levels did not associate with metabolism-related parameters, including body mass index (BMI), total cholesterol, triglycerides, low-density lipoprotein cholesterol (LDL-C), HDL-C, fasting blood glucose, and HbA1c (Supplementary Figure 1).

### Plasma SERPINA3 Levels Were Associated With CAD

**Figure 2:**
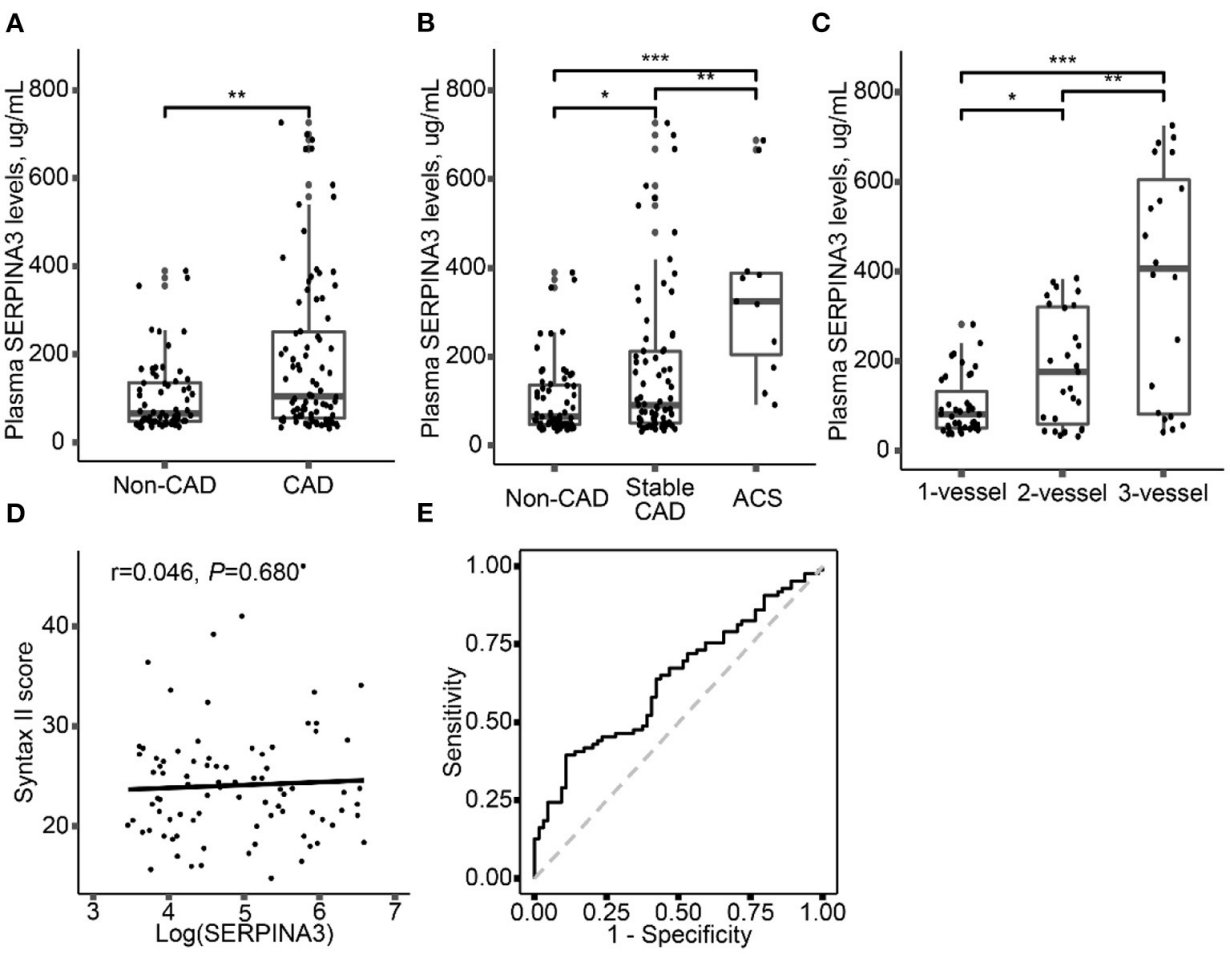
Plasma SERPINA3 levels elevated in different groups. **(A)** Comparison of plasma SERPINA3 levels in patients with CAD and non-CAD. **(B)** Comparison of plasma SERPINA3 levels in patients with non-CAD, stable CAD, and ACS. **(C)** Stratified analysis was performed by the number of diseased vessels. **(D)** Association between log-transformed plasma SERPINA3 levels and Syntax II score. **(E)** Receiver operating characteristic (ROC) curves confirmed that plasma SERPINA3 levels significantly differentiated CAD patients. Area under the curve (AUC) = 0.64, 95% CI:0.55–0.73, *P* = 0.004 for the significant AUC. *P < 0.05, **P < 0.01, ***P < 0.001.

The level of plasma SERPINA3 was significantly higher in CAD group [104.4(54.5–259.2) μg/mL] than that in non-CAD group [65.3(47.5–137.3) μg/mL] (*P* = 0.004) (Figure 2A). Plasma SERPINA3 levels were significantly higher in ACS and stable CAD patients than in non-CAD patients and were highest in ACS patients [non-CAD: 65.3(47.7–137.3) μg/mL; stable CAD: 90.9(50.8–212.4) μg/mL; ACS: 324.6(204.8–388.3) μg/mL] (Figure 2B). Of the 86 CAD patients, 39 had 1-vessel disease, 27 had 2-vessel disease and 20 had 3-vessel disease, the plasma SERPINA3 levels increased progressively with the severity of CAD [1-vessel: 80.5(50.8–131.9) μg/mL; 2-vessel:175.4(59.3–321.4) μg/mL; 3-vessel:405.9(82.0–604.9) μg/mL] (Figure 2C, P for trend < 0.001). Unfortunately, no correlation was observed between log-transformed plasma SERPINA3 levels and SYNTAX score II (Figure 2D, *P* = 0.680).

Univariate and multivariate logistic regression analysis was performed to determine the association between plasma SERPINA3 levels and CAD. As shown in Table 1, log-transformed SERPINA3 levels was independently correlated to the higher risk of CAD, when unadjusted (OR = 1.95, 95% CI: 1.27–2.98, *P* = 0.002), adjusted for Model1 (OR = 2.15, 95% CI: 1.29–3.57, *P* = 0.003) and adjusted for Model 2 (OR = 2.44, 95% CI: 1.33–4.51, *P* = 0.004). Plasma SERPINA3 levels were divided into three groups by tertiles to demonstrate this relationship further. Even fully adjusted, the tertiles of SERPINA3 levels were also significantly associated with CAD (Tertile 3 vs. Tertile 1: OR = 4.32, 95% CI:1.44–13.01, *P* = 0.009; Tertile 2 vs. Tertile 1: OR = 1.15, 95% CI:0.40–3.33, *P* = 0.790). Furthermore, we performed ROC analysis to assess the ability of plasma SERPINA3 levels to identify CAD patients from non-CAD patients, and the area under the ROC curve (AUC) was relatively low (AUC = 0.64, 95% CI: 0.55–0.73; Figure 2E).

**Table 1:** Associations of plasma SERPINA3 levels with the presence of CAD.

	**Unadjusted OR**	***P*-value**	**Adjusted for Model 1 OR**	***P*-value**	**Adjusted for Model 2 OR**	***P*-value**
Log SERPINA3	1.95 (1.27–2.98)	0.002	2.15 (1.29–3.57)	0.003	2.44 (1.33–4.51)	0.004
SERPINA3						
Tertile 1	1.00 (Reference)	–	1.00 (Reference)	–	1.00 (Reference)	–
Tertile 2	1.27 (0.58–2.79)	0.549	1.34 (0.55–3.24)	0.519	1.15 (0.40–3.33)	0.790
Tertile 3	3.34 (1.44–7.75)	0.005	4.11 (1.58–10.73)	0.004	4.32 (1.44–13.01)	0.009

*Model 1: adjusted for age, sex, diabetes mellitus, hypertension, dyslipidemia, and smoking; Model 2: adjusted for Model 1 + BMI, CRP, Cr, LDL-C, HbA1c, and LVEF. BMI, body mass index; CRP, C-reactive protein; Cr, creatinine; LDL-C, low-density lipoprotein cholesterol; HbA1c, glycated hemoglobin A1c; LVEF, left ventricular ejection fraction*.

### Secreted SERPINA3 Regulated the Release of Inflammatory Factors in HUVECs

**Figure 3:**
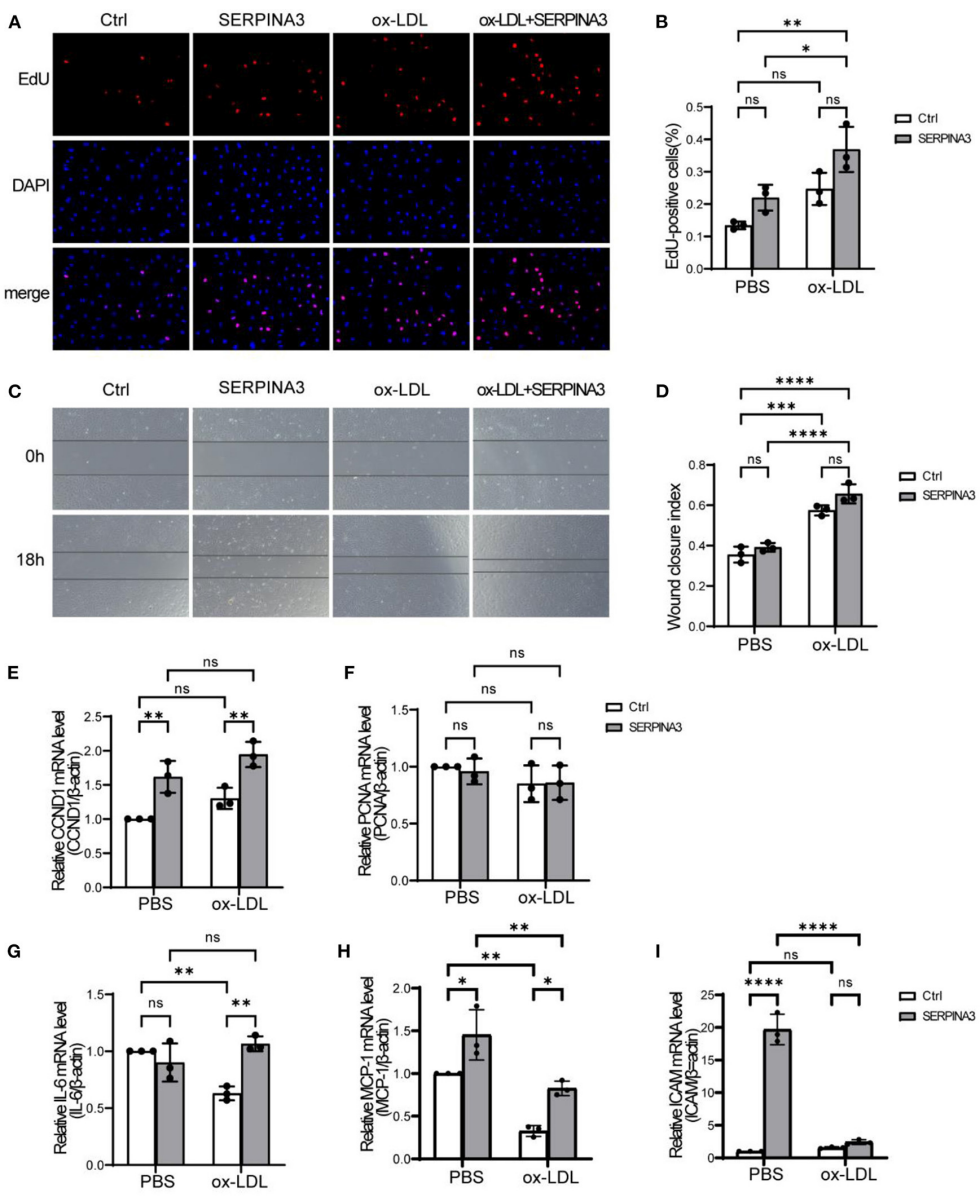
Secreted SERPINA3 regulated the release of inflammatory factors in HUVECs. **(A)** HUVECs were treated with SERPINA3 (100 ng/mL) and ox-LDL (100 μg/mL) for 18 h, cells proliferation in different groups was determined by EdU assay. **(B)** Quantification of RASMCs proliferation rates. Data are mean ± SD. *n* = 3 independent experiments, two-way ANOVA with Bonferroni post-test, ns ≥ 0.05, *P < 0.05, **P < 0.01. **(C)** HUVECs migration in different groups was determined by scratch assays at 0 and 12 h after wounding. **(D)** Quantification of RASMCs wound closure index, namely the distance migrated at time 0 relative to the distance at 12 h. Data are mean ± SD. *n* = 3 independent experiments, two-way ANOVA with Bonferroni post-test, ns ≥ 0.05, ***P < 0.005, ****P < 0.001. **(E–I)** qPCR was performed for PCNA, CCND1, IL-6, MCP-1, and ICAM of HUVECs in different groups. Data are mean ± SD. *n* = 3 independent experiments, two-way ANOVA with Bonferroni post-test, ns ≥ 0.05, *P < 0.05, **P < 0.01, ****P < 0.001.

As is well-known, endothelial cells (ECs) line up in a thin layer, forming vascular endothelium to demarcate the circulating blood and underlying tissues. ECs thus serve as “first responders” in the interaction with circulating macromolecules (19). So, we tested whether SERPINA3 can impact HUVECs or not. EdU assay and scratch assay for human SERPINA3 stimulated HUVECs showed that SERPINA3 did not enhance cells proliferation (Figures 3A,B) and migration (Figures 3C,D) obviously whether in PBS group or ox-LDL group. However, the effect of SERPINA3 in the combination of ox-LDL was statistically significant compared to it alone. qPCR results showed SERPINA3 elevated mRNA level of Cyclin D1 (CCND1) in HUVECs (Figure 3E) though proliferation cell nuclear antigen (PCNA) barely changed (Figure 3F). As for the effects on the release of other inflammatory factors, IL-6 increased after co-stimulation of SERPINA3 and ox-LDL (Figure 3G). Monocyte chemoattractant protein-1 (MCP-1) and intercellular cell adhesion molecule-1 (ICAM-1)were enhanced in various degrees (Figures 3H,I), suggesting that SERPINA3 can promote the adhesion of inflammatory cells to the lesion.

### SERPINA3 Was Expressed by Smooth Muscle Cells in Aorta

**Figure 4:**
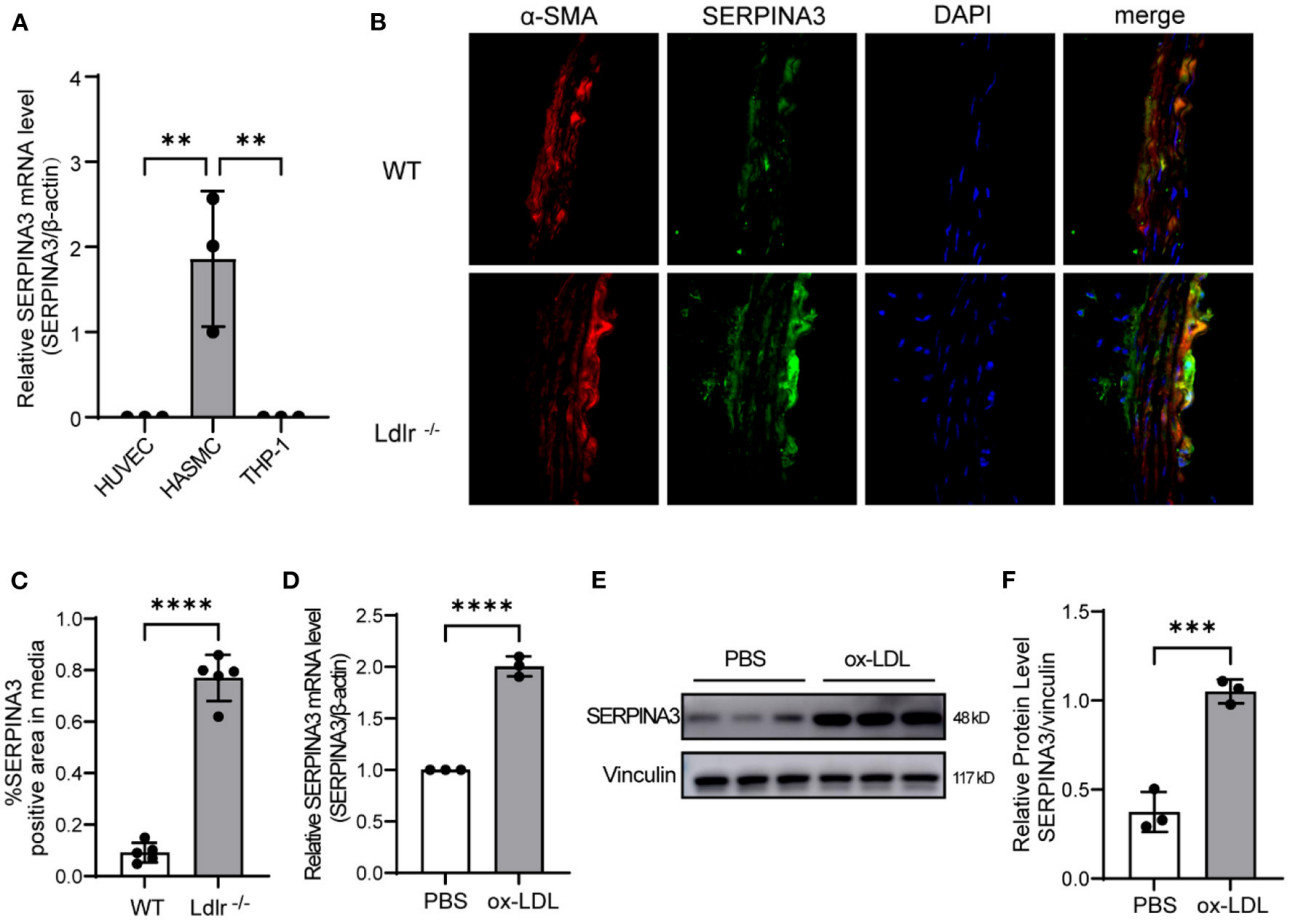
SERPINA3 expressed by smooth muscle cells in aorta. **(A)** SERPINA3 mRNA level was highest in HASMCs compared to HUVECs and THP-1. Data are mean ± SD. *n* = 3 independent experiments, one-way ANOVA with Bonferroni post-test, **P < 0.05. **(B)** Immunofluorescence staining showed that SERPINA3 (green) was upregulated in smooth muscle cells (SMCs, stained in red) residing in plaque. **(C)** Quantitative analysis of the percentages of SERPINA3-positive stained SMCs in coronary arteries (*n* = 5 per group). Normal IgG isotype serves as a negative control. Data are mean ± SD. *n* = 3 independent experiments, Unpaired Student's *t*-test, ****P < 0.001. **(D)** qPCR and **(E)** western blot were performed for SERPINA3 of RASMCs stimulated with ox-LDL. **(F)** Quantification of E. Data is mean ± SD. *n* =3 independent experiments, Unpaired Student's *t*-test, ***P < 0.01, ****P < 0.001.

It was reported that the SERPINA3 mRNA level was 14-folds higher in the plaque aorta of *Apoe*^−/−^ mice than in plaque-free aorta of *Apoe*^−/−^ mice or C57BL/6 mice (16). However, the primary cells expressing SERPINA3 in aorta remained unknown. Therefore, we tested mRNA levels of SERPINA3 in the three most common cells in aorta, and smooth muscle cells were found to have the highest expression while endothelial cells and inflammatory cells barely expressed SERPINA3 (Figure 4A). Meanwhile, immunofluorescence staining showed colocalization of SERPINA3 and α-SMA (a marker of SMC), and SERPINA3 was more expressed in *Ldlr*^−/−^ mice's aorta compared to WT mice's aorta (Figures 4B,C). Ox-LDL stimulated RASMCs, as an *in vitro* model for atherosclerosis, were detected to express more SERPINA3 in mRNA level (Figure 4D) and protein level (Figures 4E,F).

### The Knockdown of SERPINA3 Attenuated the Proliferation and Migration of RASMCs

**Figure 5:**
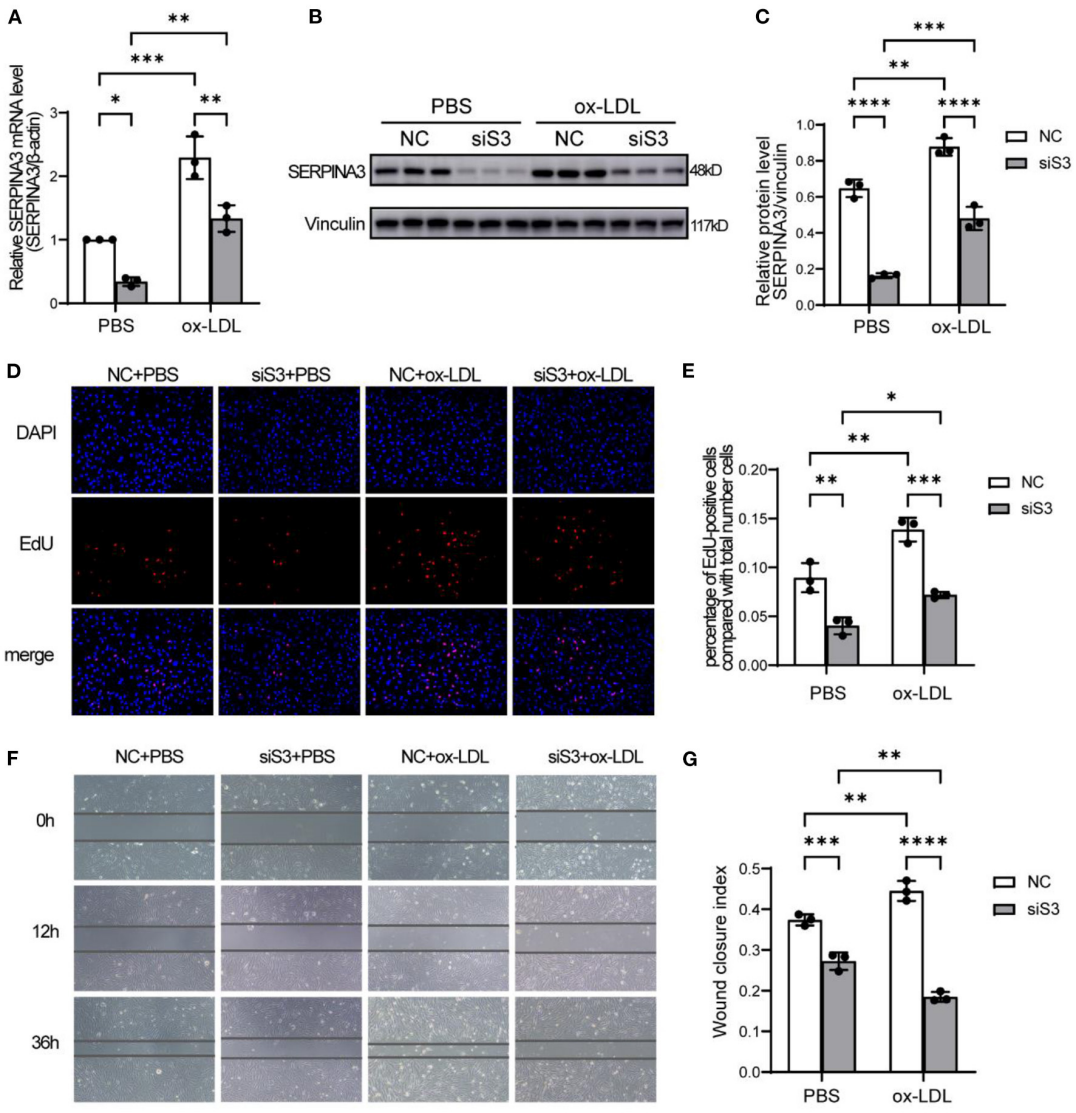
SERPINA3 silencing attenuates the proliferation and migration of RASMCs. **(A)** RASMCs were transferred SERPINA3 siRNA for 36 h and stimulated by ox-LDL for 12 h, qPCR and **(B)** western blot were performed for SERPINA3 mRNA and protein level. **(C)** Quantification of **(B)**. Data are mean ± SD. *n* = 3 independent experiments, two-way ANOVA with Bonferroni post-test, *P < 0.05, **P < 0.01, ***P < 0.005. **(D)** RASMCs proliferation in different groups was determined by EdU assay, with EdU positive cells being expressed as a percentage of the total number of cells, determined by Hoechst 33342 nuclei staining, in each field. **(E)** Quantification of RASMCs proliferation rates. Data are mean ± SD. *n* = 3 independent experiments, two-way ANOVA with Bonferroni post-test, *P < 0.05, **P < 0.01, ***P < 0.005. **(F)** RASMCs migration in different groups was determined by scratch assays at 0, 12, and 36 h after wounding. **(G)** Quantification of RASMCs wound closure index, namely the distance migrated at time 0 relative to the distance at 36 h. Data are mean ± SD. *n* = 3 independent experiments, two-way ANOVA with Bonferroni post-test, **P < 0.01, ***P < 0.005, ****P < 0.001.

To clarify the function of SERPINA3 in atherosclerosis, we used siRNA oligomer and ox-LDL in RASMCs. The efficiency of siRNA achieved a nearly 60% decrease in expression in mRNA (Figure 5A) and protein levels (Figures 5B,C). As expected, ox-LDL groups exhibited higher proliferation quantified by EdU staining compared to PBS groups. SERPINA3 knockdown groups showed lower proliferation capacity than the negative control group (Figures 5D,E).

A similar trend was observed in the scratch assay. After ox-LDL stimulation, the ability of siSERPINA3 to reduce migration was more obvious than in the PBS groups (Figures 5F,G).

### SERPINA3 Regulated Proliferation and Inflammation by NF-κB Signaling in RASMCs

**Figure 6:**
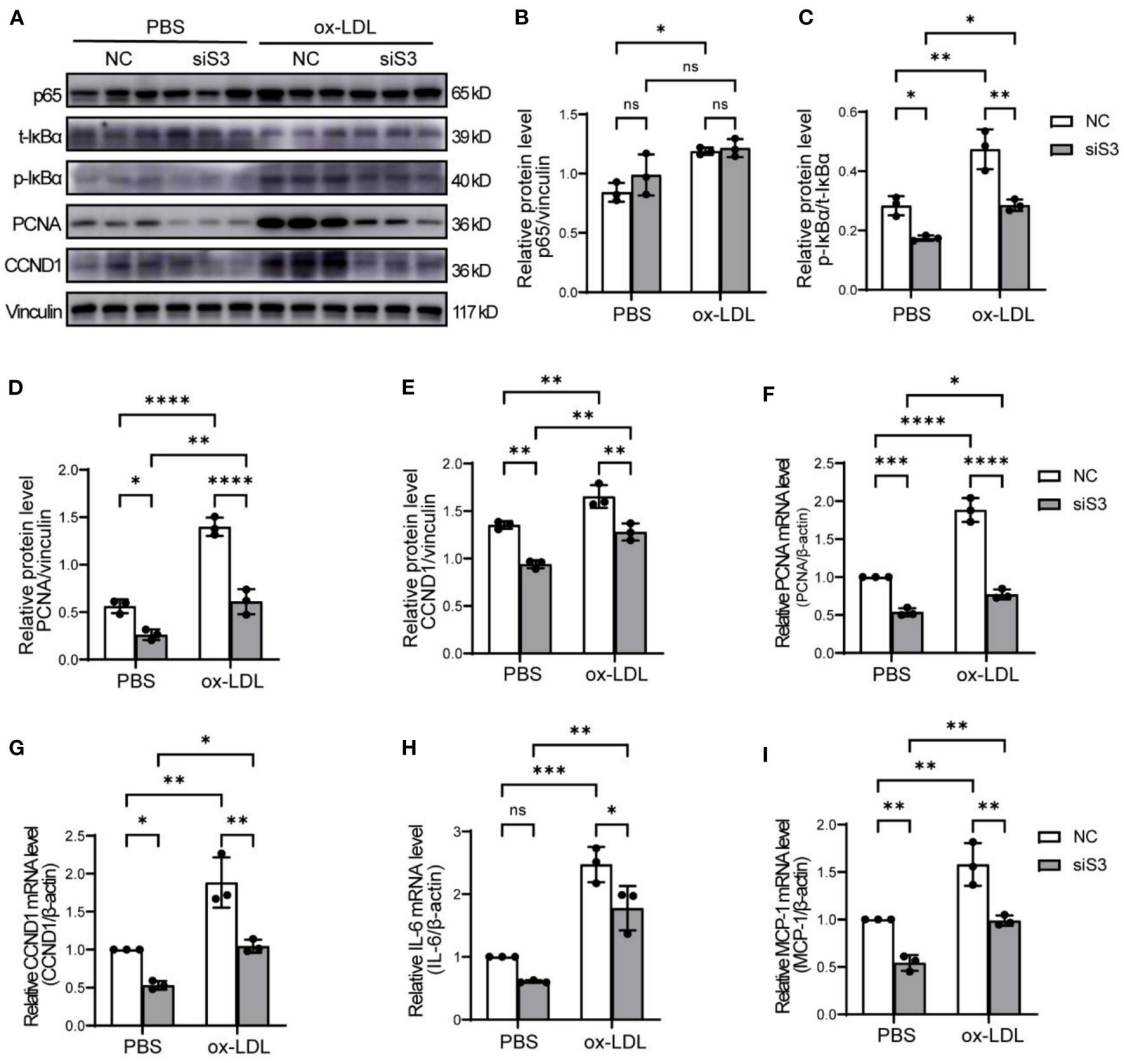
SERPINA3 activated proliferation and migration by NF-κB in RASMCs. **(A)** RASMCs was transferred SERPINA3 siRNA for 36 h and stimulated by ox-LDL for 12 h, protein expression of p65, t-IκBα, p- IκBα, PCNA, CCND1, and vinculin were measured by western blot. **(B–E)** Quantification of relative protein level of p65, p- IκBα, PCNA, CCND1 in **(A)**. Data are mean ± SD. *n* = 3 independent experiments, two-way ANOVA with Bonferroni post-test, ns ≥ 0.05, *P < 0.05, **P < 0.01, ****P < 0.001. **(F–I)** qPCR was performed for PCNA, CCND1, IL-6, and MCP-1 of RASMCs in different groups. Data are mean ± SD. *n* = 3 independent experiments, two-way ANOVA with Bonferroni post-test, ns ≥ 0.05, *P < 0.05, **P < 0.01, ***P < 0.005, ****P < 0.001.

It is generally acknowledged that NF-κB signaling is an important pathway regulating cell proliferation and inflammation. We first analyzed the protein level of p65, which could bind to the promoter regions of target genes and mediates their transcription, and found that p65 was up-regulated by ox-LDL but not influenced by knockdown of SERPINA3 in RASMCs (Figures 6A,B). But phosphorylated IκBα, which can promote the activation of NF-κB, was enhanced by ox-LDL and decreased by siSERPINA3, as total IκBα had no noticeable change in different treatment (Figures 6A,C). We next examined NF-κB downstream factors, like PCNA and CCND1 as proliferation factors, and IL-6 and MCP-1 as inflammatory factors. Western blot and qPCR results showed that the change of PCNA and CCND1 were nearly consistent with the phenotype of RASMCs (Figures 6A,D–G). In terms of inflammatory factors changes, exposure of RASMCs to ox-LDL markedly enhanced the ability of proinflammatory compared with PBS-exposed control cells, which is in accordance with theory. However, the transfection of siSERPINA3 resulted in marked inhibition of IL-6 and MCP-1 expression in mRNA (Figures 6H,I).

## Discussion

In this study, we evaluated the association between plasma SERPINA3 levels and CAD and explored the mechanism of SERPINA3 in the pathogenesis of atherosclerosis. We found that patients with CAD had higher plasma SERPINA3 levels, and elevated plasma SERPINA3 levels were associated with CAD. Secreted SERPINA3 stimulated the expression of inflammatory factors in HUVECs. SERPINA3 expressed by RASMCs promoted cell proliferation, migration, and expression of inflammatory cytokines itself by NF-κB signaling pathways.

As a member of the serpin superfamily, SERPINA3 is believed to regulate inflammation (20), mainly via inhibiting neutrophil cathepsin G (21) and uniquely binding DNA. It was reported that SERPINA3 contributes to a wide range of diseases, such as chronic obstructive pulmonary disease, Parkinson's disease, Alzheimer's disease, and cancer (13, 20). SERPINA3 levels were reported to increase in chronic heart failure patients (HF), though without prognostic value (22). Elevated expression of SERPINA3 was reported in human atherosclerotic lesions (16). The iTRAQ/MRM methods revealed that SERPINA3 could be used to distinguish patients with AMI (23). In a recent study, the investigators highlighted that patients with AMI had higher plasma SERPINA3 levels compared with healthy control (24). However, little is known about plasma SERPINA3 levels in patients with CAD. In this study, we further confirmed that plasma SERPINA3 levels were higher in patients with CAD than patients with CAG (-), and plasma SERPINA3 levels were positively associated with the severity of CAD, which was highest in patients with ACS and 3-vessels disease. Of note, SERPINA3 is an acute-phase reactant protein involved in the pathogenesis of inflammation (15), so the plasma SERPINA3 levels were significantly higher in ACS patients than in stable CAD patients. In addition, the plasma SERPINA3 levels of ACS patients measured in this study were similar to those measured in AMI patients from Zhao, L et al.' study (24), which confirmed the stability of our measurement. It is well-accepted that atherosclerosis is a chronic inflammatory disease that injuries the arterial wall.

We also found that log-transformed plasma SERPINA3 levels were positively associated with clinical inflammation indexes including neutrophils, CRP levels, and NLR, although classical inflammatory cytokines (such as IL-1, IL-6, etc.) were not measured in the patients' plasma. Based upon these, it was hypothesized that SERPINA3 may act as a pro-inflammatory effect and contribute to atherosclerosis.

Zhao, L et al. demonstrated that elevated plasma SERPINA3 levels could predict long-term MACE in patients with AMI (24). Another study also reported that plasma SERPINA3 levels might be a biomarker of chronic HF but without prognostic value (22). Whether SERPINA3 can be used as a novel diagnostic biomarker for CAD has not been investigated. Thus, we conducted the multivariate logistic regression analysis and ROC analysis. After fully adjusted, log-transformed plasma SERPINA3 levels and tertiles of those were independently associated with CAD. The ROC curve was significant but relatively low AUC [0.64 (0.55–0.73)]. Although the diagnostic value of plasma SERPINA3 levels for CAD was not well enough, the stable relationship between plasma SERPINA3 levels and CAD indicated that SERPINA3 played a significant role in the process of CAD.

Endothelial cells form a critical boundary of blood and underlying vessel tissue and play an essential role in atherosclerosis (25). Circulating SERPINA3 thus is thought to affect ECs first. Our results proved that the mRNA expression of IL-6, MCP-1, and ICAM were elevated after SERPINA3 treatment in HUVECs. Contrary to mRNA expression of CCND1, no statistically significant impact on HUVECs ability of proliferation and migration was identified by EdU assay and scratch assay when treated with SERPINA3. However, SERPINA3 worked more efficiently in combination with ox-LDL, verifying its ability was amplified in atherosclerosis. It is well-established that IL-6 was an important inflammatory factor, and MCP-1 and ICAM were immunocyte adhesion factors in atherosclerosis. In summary, secreted SERPINA3 may increase the expression of inflammatory factors and immunocyte adhesion factors in endothelial cells to participate in atherosclerosis.

As for the source of SERPINA3, on the one hand, SERPINA3 is secreted by the liver as reported in previous studies (13), because the atherosclerosis occurrence is often accompanied by systemic changes and is correlated with metabolic liver diseases through multiple pathophysiological mechanisms (26, 27). On the other hand, existing data showed an increased expression of SERPINA3 in atherosclerotic lesions from a minor stroke and TIA, and SERPINA3 mRNA level was higher in plaque aorta of *Apoe*^−/−^ mice than in plaque-free aorta of control mice (16). We also found SERPINA3 expressed in aorta and confirmed SMC was the primary expression cell. Besides, *in vitro* model of atherosclerosis displayed stimulation with ox-LDL increased the expression of SERPINA3 in RASMCs. As an anatomical and histological characteristic of atherosclerosis, significantly inflamed artery intima is positioned on top of the smooth muscle cell-rich medial layer (4). This further demonstrates that smooth muscle cells expressing SERPINA3 contribute to the progression of atherosclerosis.

It is well-documented that excess proliferation and migration of VSMC play an important role in forming atherosclerotic plaque to vascular injury, hyperglycemia, dyslipidemia, and inflammation (28–31). To clarify the role of SERPINA3 in atherosclerosis, we used siSERPINA3 oligomer and ox-LDL in RASMCs. We found that ox-LDL could promote RASMCs proliferation and migration ability, consistent with previous research (32). We also demonstrated that the knockdown of SERPINA3 attenuated ox-LDL-induced proliferation and migration in RASMCs. It meant SERPINA3 expressed by RASMCs could promote the proliferation and migration of RASMCs.

Considering the importance of the NF-κB signal pathway in cell proliferation and inflammation (33), we wanted to determine whether the knockdown of SERPINA3 might attenuate NF-κB activation. Indeed, the knockdown of SERPINA3 blocked NF-κB activation by disrupting the phosphorylation of IκBα and then decreased the cell proliferation factors (PCNA and CCND1) and inflammation factors (IL-6 and MCP-1) in RASMCs. Thus, in part, the pro-proliferation and pro-inflammation effects of SERPINA3 depended on activating the NF-κB signal pathway in RASMCs. Braghin et al. also reported that SERPINA3 induced TNF-alpha production and NF-κB activation in the murine N9 microglial cell line (34).

This study demonstrated that plasma SERPINA3 levels were significantly increased in patients with CAD, and SERPINA3 also played an essential role in the pathogenesis of atherosclerosis. However, there are several limitations to our study. Firstly, the small sample size and case-control design limited the generality and stability of the results. Secondly, the exact origin of elevated plasma SERPINA3 levels was not yet identified. Thirdly, we proved high plasma SERPINA3 levels were associated with CAD, but with lower diagnostic value for CAD. Though patients with previous ischemic stroke and CAD were excluded in this study, the patients with peripheral arterial disease were not evaluated, which may also account for the relative lower AUC of the ROC curve. Finally, we cannot clarify whether the effect of SERPINA3 on endothelial cells is achieved via a paracrine or endocrine way. Further studies should focus on exploring the source of plasma SERPINA3 in CAD patients and the impact of plasma SERPINA3 on atherosclerosis.

## Conclusion

Elevated plasma SERPINA3 levels were associated with CAD. Secreted SERPINA3 increased the expression of inflammatory cytokines and immunocyte adhesion factors in HUVECs. SERPINA3 expressed by RASMCs promoted cell proliferation, migration, and expression of inflammation cytokines in atherosclerosis via NF-κB signaling pathways in RASMCs. Therefore, SERPINA3 played an essential role in the pathogenesis of atherosclerosis.

## Data Availability Statement

The original contributions presented in the study are included in the article/Supplementary Material, further inquiries can be directed to the corresponding author/s.

## Ethics Statement

The studies involving human participants were reviewed and approved by Shanghai Tenth People's Hospital's Ethics Committee. The patients/participants provided their written informed consent to participate in this study. The animal study was reviewed and approved by Shanghai Tenth People's Hospital's Ethics Committee.

## Author Contributions

BoL and ZL contributed to the experimental study, data analysis, and manuscripts preparation of this study. YW, MZ, YZ, PJ, WK, and YS contributed to sample acquisition. BiL and XZ contributed to writing the manuscript. WP contributed to conceiving the project, designing the experiments, analyzing the data, interpreting the results, and writing the manuscript. All authors contributed to the article and approved the submitted version.

## Funding

This work was sponsored by the Clinical Research Plan of SHDC (No. SHDC2020CR4019) and the program of the National Natural Science Foundation of China (91939101 and 82070230).

## Conflict of Interest

The authors declare that the research was conducted in the absence of any commercial or financial relationships that could be construed as a potential conflict of interest.

## Publisher's Note

All claims expressed in this article are solely those of the authors and do not necessarily represent those of their affiliated organizations, or those of the publisher, the editors and the reviewers. Any product that may be evaluated in this article, or claim that may be made by its manufacturer, is not guaranteed or endorsed by the publisher.
