# Supplementary material for: The Association and Pathogenesis of SERPINA3 in Coronary Artery Disease
Source: Front Cardiovasc Med. 2021 Dec 8;8:756889. doi: 10.3389/fcvm.2021.756889 (PMC8692672; doi:10.3389/fcvm.2021.756889)
Supplement: Supplementary file 1 [file Data_Sheet_1.docx]

**Supplement data**

Table S1. Primers for Real-time PCR

| Gene Name | Primer | Sequence |
| --- | --- | --- |
| SERPINA3-human | Formate:5′-3′ | GCTCATCAACGACTACGTGAA |
|  | Reverse: 5′-3′ | CACCATTACCCACTTTTTCTTGC |
| SERPINA3-rat | Formate:5′-3′ | ACCCCTGAGACAGAAATCCACCG |
|  | Reverse: 5′-3′ | TTCAGCTCCTCATCCCGGACGTA |
| PCNA-rat | Formate:5′-3′ | AAAGGACGGGGTGAAGTTTTCTGC |
|  | Reverse: 5′-3′ | AACTGGCTCATTCATCTCTATGGACAC |
| Cyclin D1-rat | Formate:5′-3′ | GAGGCGGATGAGAACAAGCAGATC |
|  | Reverse: 5′-3′ | GGAGGGTGGGTTGGAAATGAACTTC |
| IL-6-rat | Formate:5′-3′ | ACTTCCAGCCAGTTGCCTTCTTG |
|  | Reverse: 5′-3′ | TGGTCTGTTGTGGGTGGTATCCTC |
| MCP-1-rat | Formate:5′-3′ | CACCTGCTGCTACTCATTCACTGG- |
|  | Reverse: 5′-3′ | CTTCTTTGGGACACCTGCTGCTG |
| β-actin-rat | Formate:5′-3′ | ATGGTGGGAATGGGTCAGAA |
|  | Reverse: 5′-3′ | CTTTTCACGGTTGGCCTTAG |
| β-actin-human | Formate:5′-3′ | CGCAAAGACCTGTACGCCAAC |
|  | Reverse: 5′-3′ | CACGGAGTACTTGCGCTCAGG |

| Table S2. Demographic and laboratory characteristics in CAD and non-CAD groups. | | | |
| --- | --- | --- | --- |
|  | CAD n=86 | non-CAD n=64 | *P* value |
| Age, years | 63.5±6.6 | 62.5±6.3 | 0.356 |
| Sex (Male) | 58(67.4%) | 23(35.9%) | <0.001 |
| Hypertension | 46(53.5%) | 32(50.0%) | 0.672 |
| Diabetes mellitus | 21(24.4%) | 6(9.4%) | 0.018 |
| Hyperlipidemia | 8(9.3%) | 4(6.2%) | 0.496 |
| Smoker | 28(32.6%) | 9(14.1%) | 0.009 |
| BMI, Kg/m^2^ | 24.5±3.3 | 24.2±3.5 | 0.609 |
| Type of CAD |  |  |  |
| Stable CAD | 74(86.0%) | - | - |
| ACS | 12(14.0%) | - | - |
| SBP, mmHg | 137.1±21.5 | 132.8±17.3 | 0.191 |
| DBP, mmHg | 77.1±13.2 | 73.9±9.6 | 0.110 |
| HR, bpm | 80.7±12.9 | 81.8±14.2 | 0.618 |
| Syntax I score | 7.0(5.0-12.8) | - | - |
| Syntax II score | 23.3(20.2-26.8) | - | - |
| Diseased vessel |  |  |  |
| LM | 4(4.7%) | - | - |
| LAD | 68(79.1%) | - | - |
| LCX | 41(47.7%) | - | - |
| RCA | 43(50.0%) | - | - |
| WBC, *10^9/L | 6.43±1.93 | 5.90±1.48 | 0.065 |
| Neutrophils, *10^9/L | 62.6±12.1 | 60.1±13.2 | 0.231 |
| Lymphocytes, *10^9/L | 27.2±8.9 | 28.9±9.7 | 0.284 |
| CRP, mg/L | 2.98(0.80-2.98) | 2.98(0.96-2.98) | 0.431 |
| NLR | 2.67±1.55 | 2.40±1.34 | 0.275 |
| Cr, umol/L | 70.7(55.0-84.0) | 63.9(53.9-81.5) | 0.055 |
| Uric acid, umol/L | 324.0(264.0-396.0) | 314.4(247.8-391.6) | 0.088 |
| Total cholesterol, mmol/L | 4.32(3.75-4.99) | 4.34(3.60-4.78) | 0.642 |
| Triglycerides, mmol/L | 1.48(1.00-2.15) | 1.25(0.91-1.68) | 0.054 |
| LDL-C, mmol/L | 2.68(2.25-3.28) | 2.68(1.86-3.10) | 0.524 |
| HDL-C, mmol/L | 1.03(0.86-1.27) | 1.13(0.97-1.39) | 0.011 |
| Fasting glycemia, mmol/L | 5.3(4.7-6.5) | 4.9(4.6-5.4) | 0.012 |
| HbA1c, % | 6.3(5.8-7.0) | 6.1(5.8-6.4) | 0.042 |
| LVEF, % | 63.0(60.0-64.0) | 62.5(62.0-65.0) | 0.728 |

CAD, coronary artery disease; BMI, body mass index; ACS, acute coronary syndrome; SBP, systolic blood pressure; DBP, diastolic blood pressure; HR, heart rate; LM, left main trunk; LAD, left anterior descending artery; LCX, left circumflex coronary artery; RCA, right coronary artery; WBC, white blood cell; CRP, C-reactive protein; NLR, neutrophils to lymphocytes ratio; Cr, creatinine; LDL-C, low density lipoprotein cholesterol; HDL-C, high density lipoprotein cholesterol; HbA1c, glycated hemoglobin; LVEF, left ventricular ejection fraction.

Figure S1


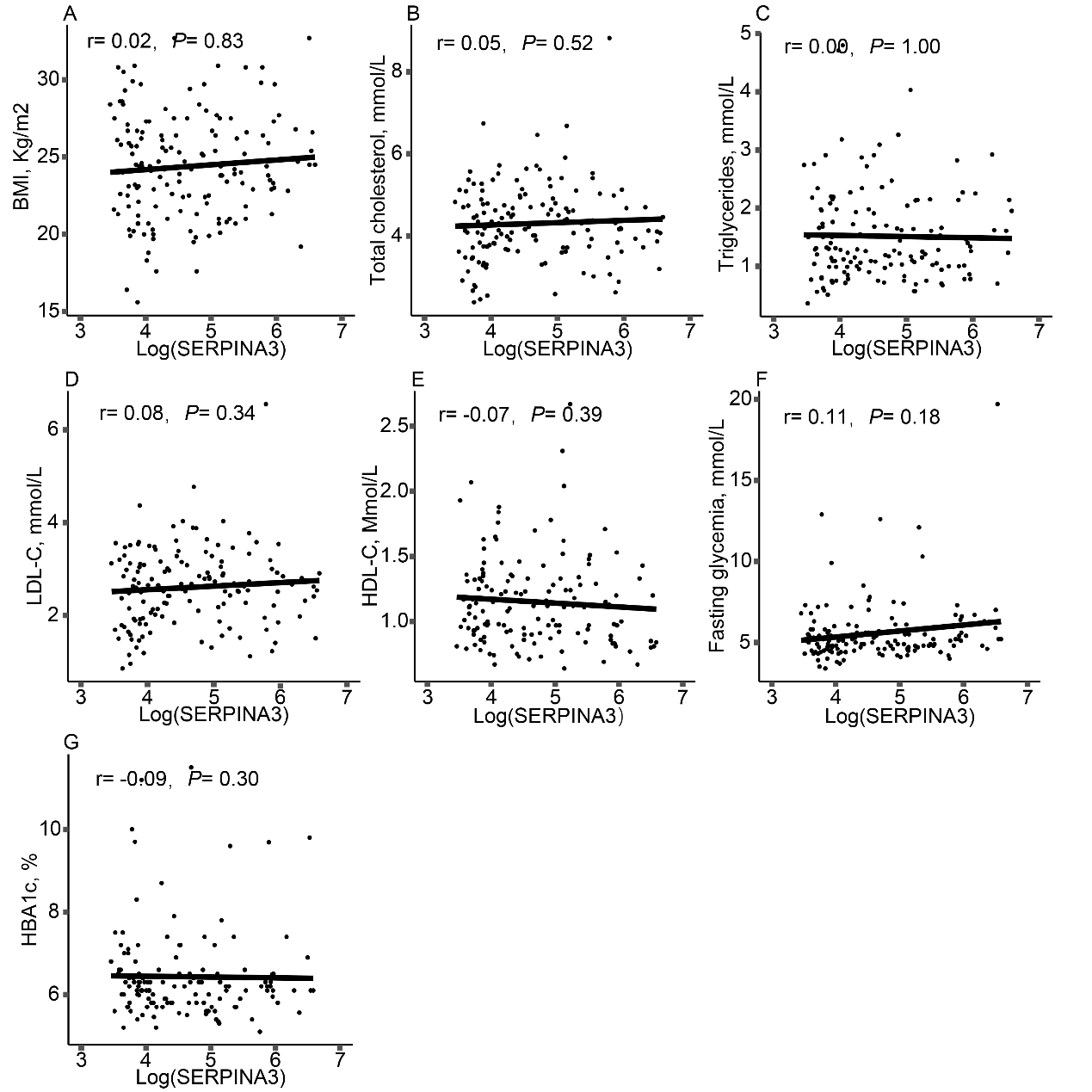


Figure S1. Associations between log-transformed plasma SERPINA3 levels with BMI (A), total cholesterol (B), triglycerides (C), LDL-C (D), HDL-C (E), fasting glycemia (F) and HbA1c (G). BMI, body mass index; LDL-C, low density lipoprotein cholesterol; HDL-C, high density lipoprotein cholesterol; HbA1c, glycated hemoglobin.
